# Laparoscopic Intraperitoneal Onlay Mesh Repair for Symptomatic Secondary Lumbar Hernia After Retroperitoneoscopic Nephrectomy: A Case Report

**DOI:** 10.7759/cureus.75922

**Published:** 2024-12-18

**Authors:** Tomoya Miura, Shingo Tsujinaka, Toru Nakano, Yu Katayose, Chikashi Shibata

**Affiliations:** 1 Gastroenterological Surgery, Tohoku Medical and Pharmaceutical University, Sendai, JPN; 2 Hepato-Biliary and Pancreatic Surgery, Tohoku Medical and Pharmaceutical University, Sendai, JPN

**Keywords:** composite mesh, laparoscopic intraperitoneal onlay mesh, lumbar hernia repair, retroperitoneal laparoscopic urological surgery, secondary lumbar hernia

## Abstract

Lumbar hernia (LH) is a rare abdominal wall hernia that occurs within the anatomic boundaries of the 12th rib, iliac crest, external oblique muscles, erector spinae muscles, and vertebral column. Secondary LH after urological surgery is rare, and the limited evidence hinders consensus on optimal surgical treatment. Here, we present a case of laparoscopic intraperitoneal onlay mesh (IPOM) repair for a large, symptomatic secondary LH after retroperitoneoscopic nephrectomy (RN) with mid-term postoperative outcomes. A 58-year-old man presented with a bulge, pain, and discomfort in the right lumbar area. Three months earlier, he had undergone RN for clear cell carcinoma of the right kidney (pT3aN0M0: stage III). Computed tomography (CT) revealed a right LH with a 10 × 7 cm orifice containing the ascending colon. Considering the symptomatic LH and associated risk of bowel obstruction, laparoscopic surgery was performed eight months after the previous RN. Laparoscopic exploration revealed a 10 (transverse) × 7 (longitudinal) cm defect in the right lateral abdominal wall, with adhesion of the ascending colon. After exposing the hernia orifice, the defect was covered using a composite mesh (Ventralight™ST, BD, Franklin Lakes, NJ, USA). The mesh was trimmed to 16 (transverse) × 13 (longitudinal) cm in size and anchored to the abdominal wall using a single, full-thickness suture. Subsequently, nonabsorbable tacks (CapSure™, BD, Franklin Lakes, NJ, USA) were applied using the double-crown technique. The postoperative course was uneventful, except for the development of a subcutaneous seroma that resolved spontaneously within four months. Follow-up CT performed 36 months after the surgery revealed a slight mesh bulge. However, the patient remained in good physical condition without recurrent symptoms, including a bulge or discomfort. Laparoscopic IPOM repair for secondary LH after RN is safe and effective in alleviating symptoms and preventing recurrence in the mid-term follow-up period. This technique simplifies surgery by avoiding re-dissection of the retroperitoneal space.

## Introduction

Lumbar hernia (LH) is a rare abdominal wall hernia that occurs within the anatomic boundaries of the 12th rib, iliac crest, external oblique muscles, erector spinae muscles, and vertebral column [1]. LH can be classified as congenital or acquired, with the latter resulting from trauma, infection, inflammation, or flank surgical incisions, such as those for nephrectomy, lumbotomy, or abdominal aortic aneurysm repair [1-4]. The overall incidence rate of incisional hernia after urological surgery is 4.8%, with lower rates observed after laparoscopic procedures (1.9%) and retroperitoneal approach (0.9%) [5]. Therefore, secondary LH occurrence after urological surgery is rare. The updated International Endohernia Society (IEHS) guidelines recommend laparoscopic repair over open repair due to reduced morbidity rates and shorter postoperative stays [6]. However, the recently published European Hernia Society (EHS) and American Hernia Society (AHS) guidelines [1] highlight the lack of consensus on optimal surgical treatment, given the scarcity of evidence. Therefore, the preferred surgical procedure for LH repair should be determined on a case-specific basis.

For secondary LH after retroperitoneoscopic nephrectomy (RN), the laparoscopic approach with intraperitoneal onlay mesh (IPOM) technique is suitable as it avoids scar dissection and mesh placement in the previously explored retroperitoneal space. Here, we present a case of laparoscopic IPOM repair for a large, symptomatic secondary LH after RN with mid-term postoperative outcomes.

## Case presentation

A 58-year-old man presented with a bulge, pain, and discomfort in the right lumbar area. Three months earlier, he had undergone RN for clear cell carcinoma of the right kidney (pT3aN0M0: stage III). The surgical wound was closed in layers using interrupted 1-0 absorbable sutures. The operative time was 337 minutes, and the estimated blood loss was 55 mL. The postoperative course was uneventful, without any significant complications. During the postoperative follow-up period, the patient developed a progressively enlarging bulge below the right lumbar oblique incision at the nephrectomy site, accompanied by increasing pain and discomfort. He was referred for surgical treatment.

**Figure 1:**
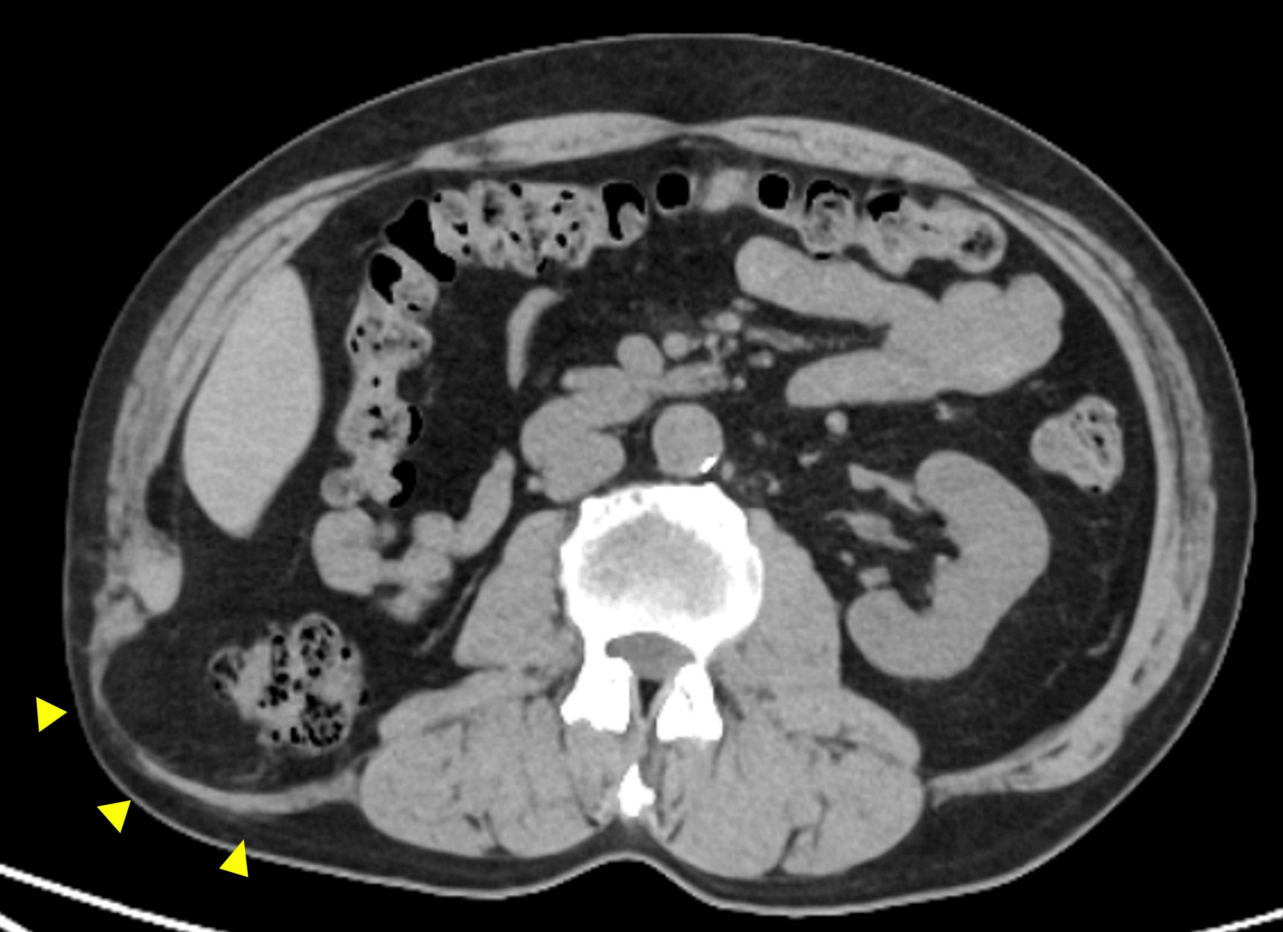
Preoperative computed tomography image of the lumbar hernia The image reveals protrusion of the hernia sac containing retroperitoneal fat tissue and the ascending colon (yellow arrowheads).

Computed tomography (CT) revealed a right lumbar hernia with a 10 × 7 cm orifice containing the ascending colon (Figure 1).

Considering the symptomatic LH and the risk of bowel obstruction, laparoscopic surgery was performed eight months after the previous RN.

**Figure 2:**
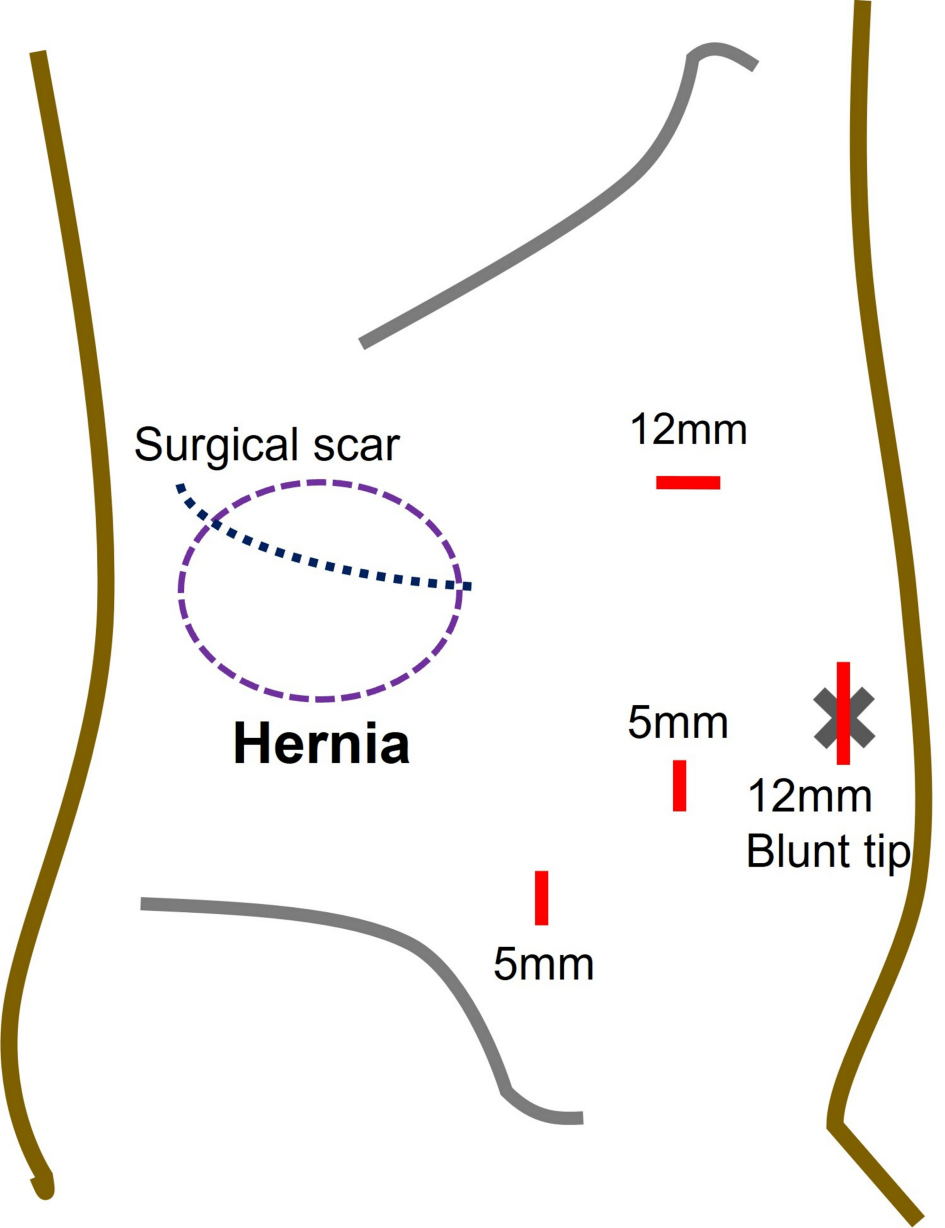
Schematic presentation of hernia location, previous surgical scar, and port setting for laparoscopic surgery Source: This is our artwork, created using Microsoft PowerPoint software (Microsoft Corp., Redmond, WA, USA).

Under general anesthesia, the patient was placed in a left-sided half-decubitus position. The port placement was performed as illustrated in Figure 2.

**Figure 3:**
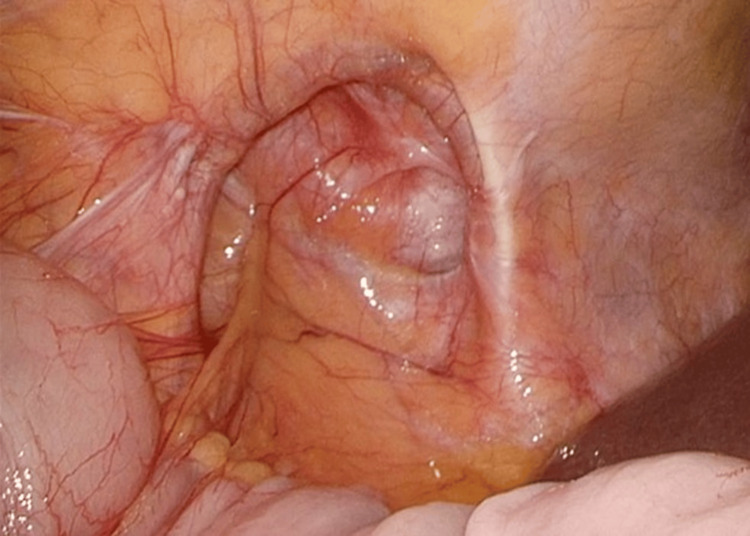
Hernia orifice

Laparoscopic exploration revealed a 10 (transverse) × 7 (longitudinal) cm defect in the right lateral abdominal wall with adhesion of the ascending colon. The hernia orifice was located medial to the Monk’s white line, bordered cranially by the costal arch and caudally by the iliac crest (Figure 3).

**Figure 4:**
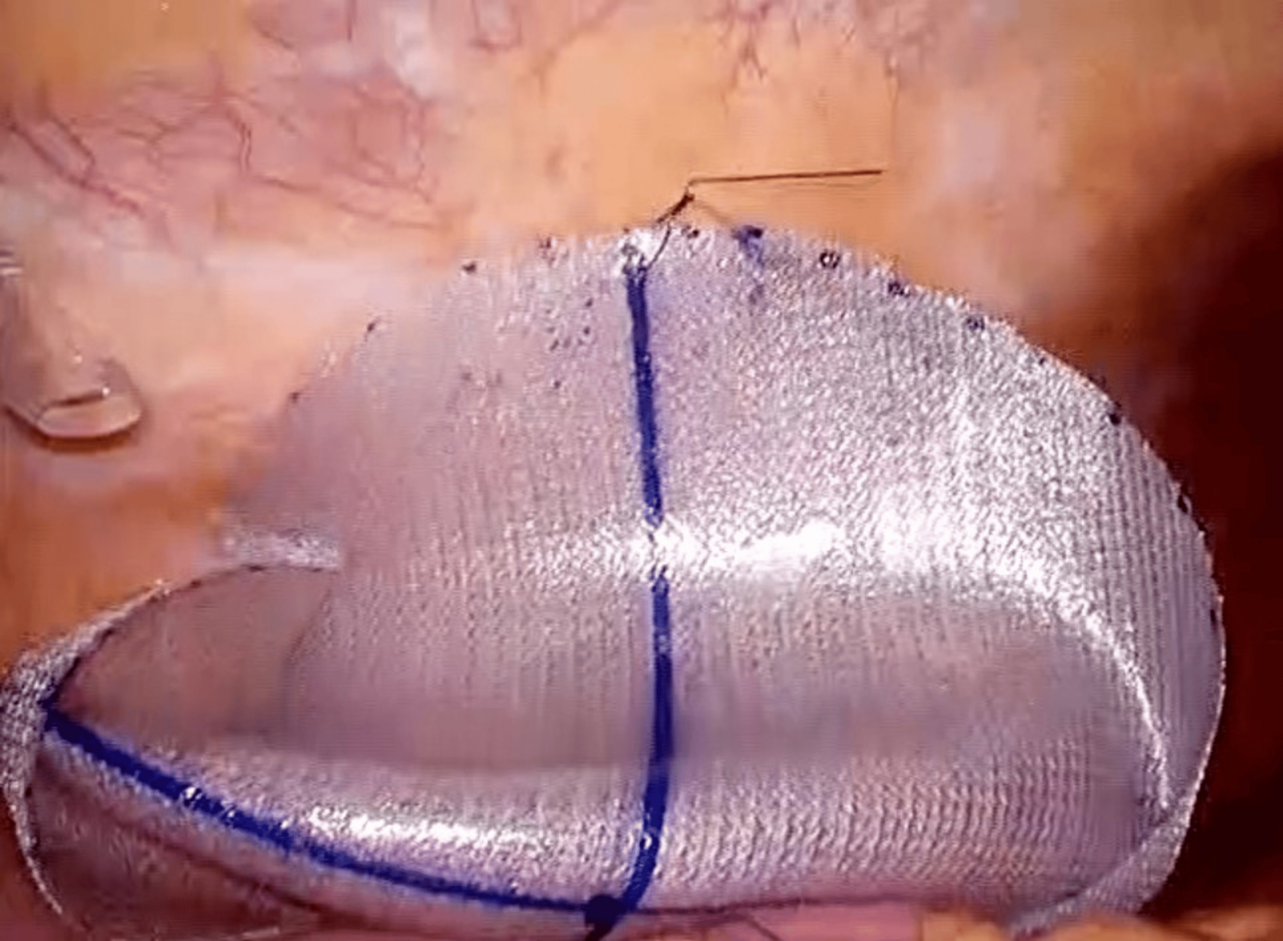
Mesh placement

The defect closure was challenging due to the large size of the defect, its proximity to the iliac crest and ribs, and the weakened abdominal wall structures. The surgical procedure began with adhesiolysis, followed by mobilization of the ascending colon toward the hepatic flexure. After exposing the hernia orifice, the defect was covered using a composite mesh (Ventralight™ ST, BD, Franklin Lakes, NJ, USA). The mesh was trimmed to 16 (transverse) × 13 (longitudinal) cm in size and anchored to the abdominal wall using a single, full-thickness suture with a 2-0 nonabsorbable material at the internal edge of the mesh (Figure 4).

**Figure 5:**
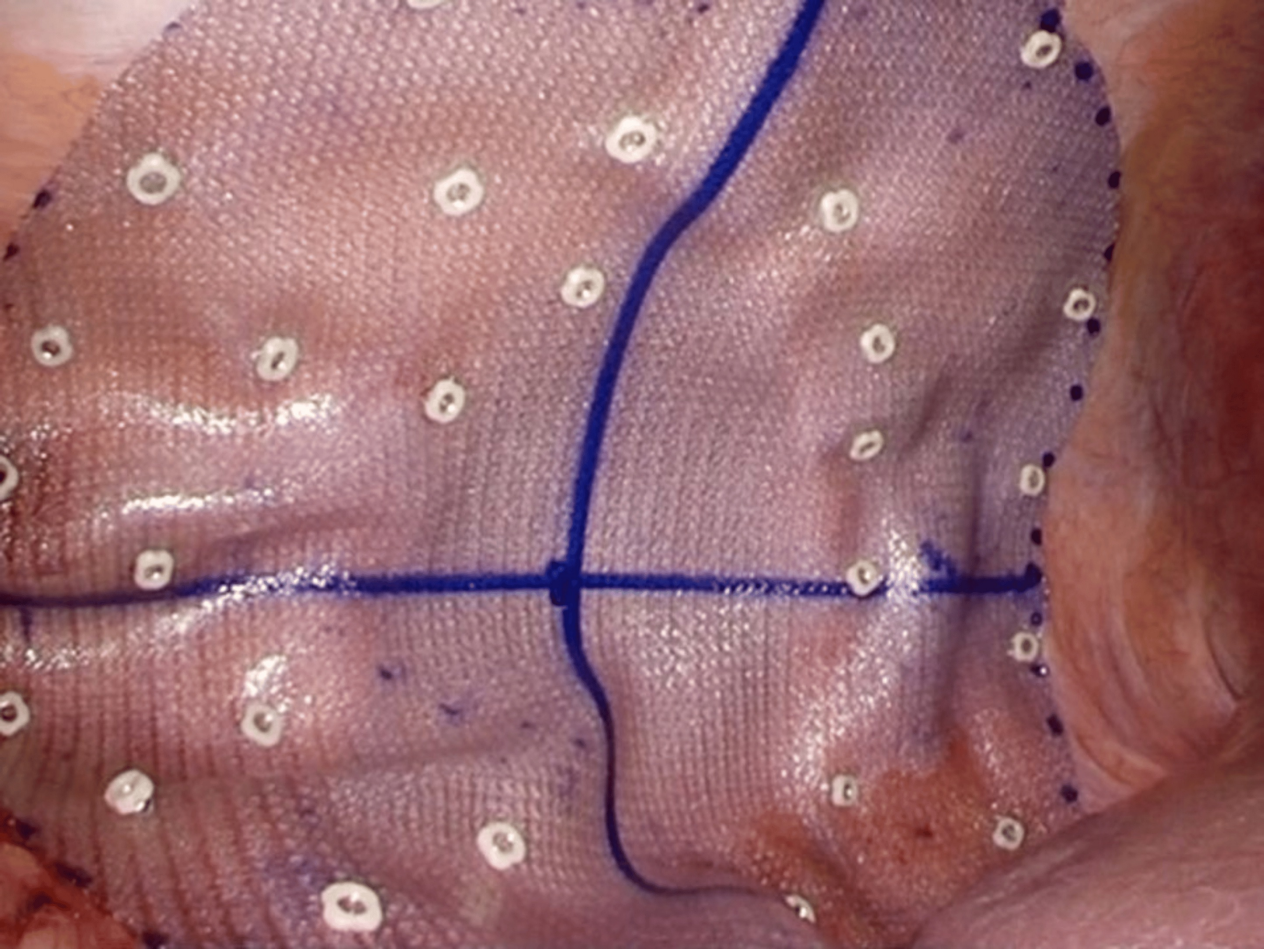
Mesh fixation

Subsequently, nonabsorbable tacks (CapSure™, BD, Franklin Lakes, NJ, USA) were applied using the double-crown technique, with careful attention to avoid injury to the intercostal arteries and nerves (Figure 5).

The operative time was 98 minutes, and the estimated blood loss was 10 mL.

**Figure 6:**
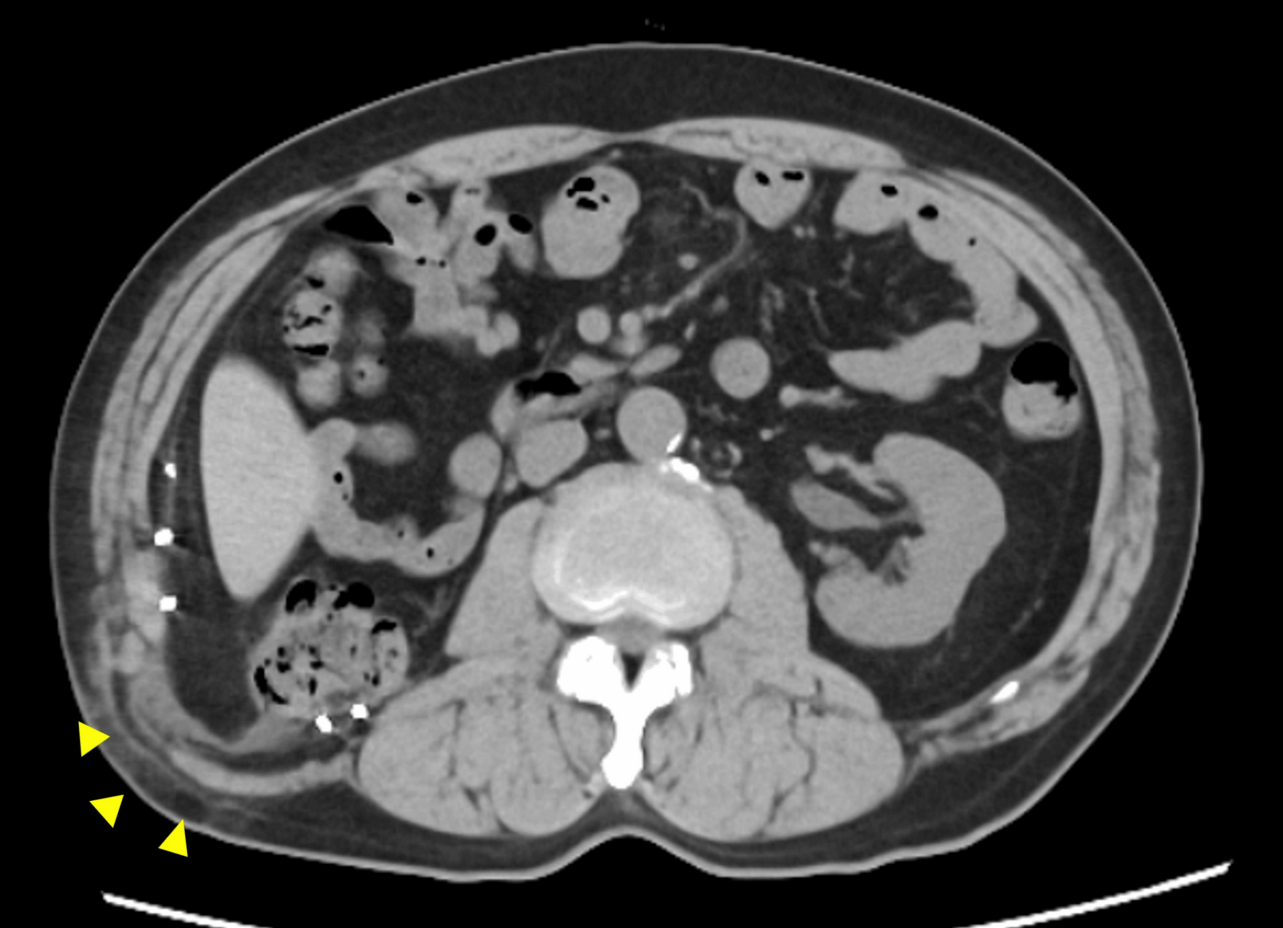
Computed tomography image obtained 36 months after the surgery A slight mesh bulge without herniation of visceral organs is observed (yellow arrowheads).

The postoperative course was uneventful, except for the development of a subcutaneous seroma that spontaneously resolved within four months. The patient was prescribed oral non-opioid analgesics (acetaminophen) for 12 days postoperatively and experienced no chronic pain. Follow-up CT performed 36 months after the surgery revealed a slight mesh bulge (Figure 6).

However, the patient remained in good physical condition without recurrent symptoms, including a bulge or discomfort.

## Discussion

The typical signs and symptoms of LH include a bulge, discomfort, and tenderness in the lumbar region [2,7]. Surgical intervention for LH is indicated based on the severity of the associated clinical indicators, including bulge, pain, gastrointestinal manifestations, worsening cosmesis, or emotional distress [3,8]. Emergency surgery is required in cases of incarcerated or strangulated LH, which occurs in only 9% of cases [8].

The common surgical approaches for LH include an open anterior approach through the previous incision, an open median approach, a laparoscopic transabdominal approach, and a retroperitoneal laparoscopic approach [3,8,9]. In the present case, we opted for a laparoscopic transabdominal approach to mobilize the ascending colon attached to the hernia sac and to avoid re-dissecting the area explored during the initial RN. The laparoscopic approach offers several technical advantages, including a comprehensive anatomic view of the entire lumbar area; easy identification of the hernia orifice, hernia type, and hernia content; avoidance of excessive dissection of the lumbar area; secure mesh reinforcement of the potentially vulnerable surrounding abdominal wall; and the ability to perform surgery via small incisions in obese patients [2,7,10]. Regarding surgical outcomes, the Herniamed registry database indicates that open sublay repair is associated with an increased risk of postoperative complications compared with laparoscopic IPOM repair [11]. However, compared with open sublay and open onlay repairs [11], laparoscopic IPOM repair is associated with an increased risk of intraoperative complications, such as iliohypogastric nerve or visceral organ injury [3,9]. Regarding hernia recurrence, large defect sizes and a high body mass index are significant risk factors. Despite this, laparoscopic IPOM repair is associated with a low risk compared with open onlay, open IPOM, and open direct suture methods, with no significant difference between laparoscopic IPOM and open sublay repairs [11].

The choice of surgical approach often depends on the size of the defect. Previous studies have shown that the laparoscopic approach is preferred for small defects, typically those less than 10 cm in size [2,3,10,11]. Amaral et al. considered laparoscopic IPOM repair alone for nonrecurrent hernias smaller than 5 cm, a combined open and laparoscopic approach for medium-sized defects (5-15 cm), and an open preperitoneal approach for large (>15 cm) and complex defects [12].

In the present case, the implanted mesh measured 16 × 13 cm, bridging a 10 × 7 cm defect. This overlap may be considered inadequate based on the EHS and AHS guidelines recommending at least a 5-cm overlap [13] or the updated IEHS guidelines, which suggest a mesh area-to-defect ratio of 16:1 [6]. However, for LH, anatomical proximity to bony structures limits wide dissection and proper mesh overlap/fixation [1,10]. Due to limited evidence, the guidelines have yet to define adequate overlap for rare hernia locations [1]. Nevertheless, in some case series, at least a 5-cm overlap was achieved for defects of small or comparable sizes to the present case (Giacosa et al. [2], 47.7 cm^2^; Edwards et al. [14], 188 cm^2^). These findings emphasize the need for technical advancements, particularly in achieving proper placement for large meshes.

In the present case, the regional bulge remains at the oblique incisional site after hernia repair, although the patient is asymptomatic and has experienced no hernia recurrence. The bulge is likely attributable to paralysis, denervation, and attenuation of the musculoaponeurotic layers caused by the initial nephrectomy, rather than the hernia repair itself [3,12]. To minimize postoperative bulging, we suggest performing defect closure with nonabsorbable sutures in addition to mesh implantation (IPOM-Plus). However, this approach may be challenging in the laparoscopic repair of secondary LH with large defects due to the inability of suture approximation to adequately support increased intra-abdominal pressure, particularly when adjacent tissue is scarred or weakened. Additionally, tight defect closure contradicts the principle of tension-free hernia repair and may lead to recurrence caused by mesh extrusion into the defect [12].

Previous reports revealed that IPOM-Plus was performed in 90% of cases with a mean hernia width of 5.8 cm [2], and defect closure was achieved in all cases with a mean hernia width of 6.4 cm using combined open and laparoscopic approaches [12]. Thus, defect closure can be considered when the adjacent tissue has sufficient strength to be secured without excessive tension.

Alternative surgical techniques to laparoscopic IPOM repair for primary or secondary LH, including transabdominal preperitoneal repair [7,15], retroperitoneal totally endoscopic prosthetic repair [16], combined open and laparoscopic approaches [12], and single-incision retroperitoneal laparoscopic repair [9], have demonstrated favorable surgical outcomes. Additionally, self-gripping mesh has been introduced in laparoscopic LH repair to reduce postoperative pain and minimize nerve or vascular injuries by eliminating the need for tackers or full-thickness anchor sutures for mesh fixation [9]. Future studies are necessary to evaluate the long-term outcomes of these emerging techniques and prosthetic materials.

## Conclusions

Laparoscopic IPOM repair for secondary LH after RN is a safe and effective procedure for reducing symptoms and minimizing hernia recurrence during the mid-term follow-up period. Although emerging evidence highlights the potential of innovative endoscopic techniques, laparoscopic IPOM repair remains a viable standard procedure for secondary LH after RN due to its straightforward technique, shorter operative time, and avoidance of retroperitoneal re-dissection.
